# Drying and temperature induced conformational changes of nucleic acids and stallion sperm chromatin in trehalose preservation formulations

**DOI:** 10.1038/s41598-021-93569-y

**Published:** 2021-07-07

**Authors:** Raffaele Brogna, Juezhu Fan, Harald Sieme, Willem F. Wolkers, Harriëtte Oldenhof

**Affiliations:** 1 Biostabilization Laboratory, Lower Saxony Centre for Biomedical Engineering, Implant Research and Development, University of Veterinary Medicine Hannover, Hannover, Germany; 2 Unit for Reproductive Medicine, Clinic for Horses, University of Veterinary Medicine Hannover, Bünteweg 15, 30559 Hannover, Germany

**Keywords:** Biotechnology, Animal biotechnology

## Abstract

Even though dried sperm is not viable, it can be used for fertilization as long as its chromatin remains intact. In this study, we investigated drying- and temperature-induced conformational changes of nucleic acids and stallion sperm chromatin. Sperm was diluted in preservation formulations with and without sugar/albumin and subjected to convective drying at elevated temperatures on glass substrates. Accumulation of reactive oxygen species was studied during storage at different temperatures, and the sperm chromatin structure assay was used to assess DNA damage. Fourier transform infrared spectroscopy was used to identify dehydration and storage induced conformational changes in isolated DNA and sperm chromatin. Furthermore, hydrogen bonding in the preservation solutions associated with storage stability were investigated. Reactive oxygen species and DNA damage in dried sperm samples were found to accumulate with increasing storage temperature and storage duration. Non-reducing disaccharides (i.e., trehalose, sucrose) and albumin counteracted oxidative stress and preserved sperm chromatin during dried storage, whereas glucose increased DNA damage during storage. When sperm was dried in the presence of trehalose and albumin, no spectral changes were detected during storage at refrigeration temperatures, whereas under accelerated aging conditions, i.e., storage at 37 °C, spectral changes were detected indicating alterations in sperm chromatin structure.

## Introduction

Cryopreservation of sperm is widely used for preservation of male fertility and assisted reproductive technologies. Maintenance of cryopreserved specimens, however, requires bulky liquid nitrogen storage containers. Storage of specimens in the dried state at refrigerated or ambient temperatures would simplify shipment, and reduce the costs and carbon footprint that are inherent to storage in liquid nitrogen. Furthermore, it would serve as an attractive alternative method for genome resource banking, which can also be applied in underdeveloped countries or remote locations with limited infrastructure^1−3^. Whereas cryopreservation generally yields viable and fully functional cells, convective drying or freeze-drying does not. Dry preservation of cells that lack adaptive mechanisms remains a challenge^4,5^. Nonetheless, nuclei obtained from dried non-viable cells have been successfully used for generating viable offspring by somatic nuclear transfer^6,7^ and intracytoplasmic sperm injection into an oocyte^8–10^.

Male fertility and success rates for producing offspring correlate with sperm chromatin structure and DNA integrity^11,12^, as well as the extent of oxidative stress the cells have been exposed to^13^. Storage stability of chromatin in dried sperm in turn is affected by the storage temperature and duration^14,15^, the composition of the preservation solution^16−19^, the residual sample water content^20,21^ and the atmospheric storage conditions^22^. Also, the inherent sperm maturation/disulfide status affects tolerance towards dehydration-induced damage^17^.

During sperm maturation histones are replaced by protamines, leading to an increased formation of disulfide bonds and tightly packed chromatin structure^23,24^. DNA of ejaculated sperm is more condensed compared to chromatin in epididymal sperm or somatic cells, rendering it relatively resistant towards external stimuli including drying^25−27^ and sub-optimal storage^19,28^. Loosely packed chromatin is more susceptible to oxidative DNA damage^29^. In eukaryotic cells, DNA can assume different helical conformations. The transition from the B- to A-like DNA form has been suggested to play a role in resistance to extreme conditions, e.g., in microorganisms and viruses, and A-DNA is typically formed under relative low water content^30,31^. The B- to A-DNA form transition involves conformational changes in the major and minor groove widths, base parameters, sugar pucker and torsional angles^32^.

Although water is typically required for the living state, so-called anhydrobiotic organisms can withstand almost complete dehydration^33−36^. These organisms can remain in a state of suspended animation at ambient conditions because they accumulate specific disaccharides and stress proteins in response to drying. Upon drying, sugars are involved in replacing hydrogen bonding interactions between biomolecules and water, as well as formation of a vitrified/glassy state in which molecular mobility and damaging reactions are slowed down^33,37^. Proteins may scavenge reactive oxygen species and increase the glass transition temperature and stability of dried specimens^28,36,38^.

In this study, we investigated drying and temperature induced conformational changes of nucleic acids and sperm chromatin. Special emphasis was placed on developing a simple method to preserve stallion sperm chromatin structure during dried storage and using infrared spectroscopy for quality assessment. Sperm was diluted in preservation formulations with and without sugar/albumin, and subjected to fast convective drying at elevated temperature to facilitate formation of a protective glassy state. Accumulation of reactive oxygen species (ROS) in dried specimens was studied during storage at different temperatures, and the sperm chromatin structure assay was used to assess DNA damage. In addition, Fourier transform infrared spectroscopy was used to identify characteristic spectral markers for B- and A-DNA, and conformational changes in sperm associated with storage stability and chromatin structure preservation.

## Results

### Effects of drying temperature/rate on the appearance of droplets of different compositions

**Figure 1:**
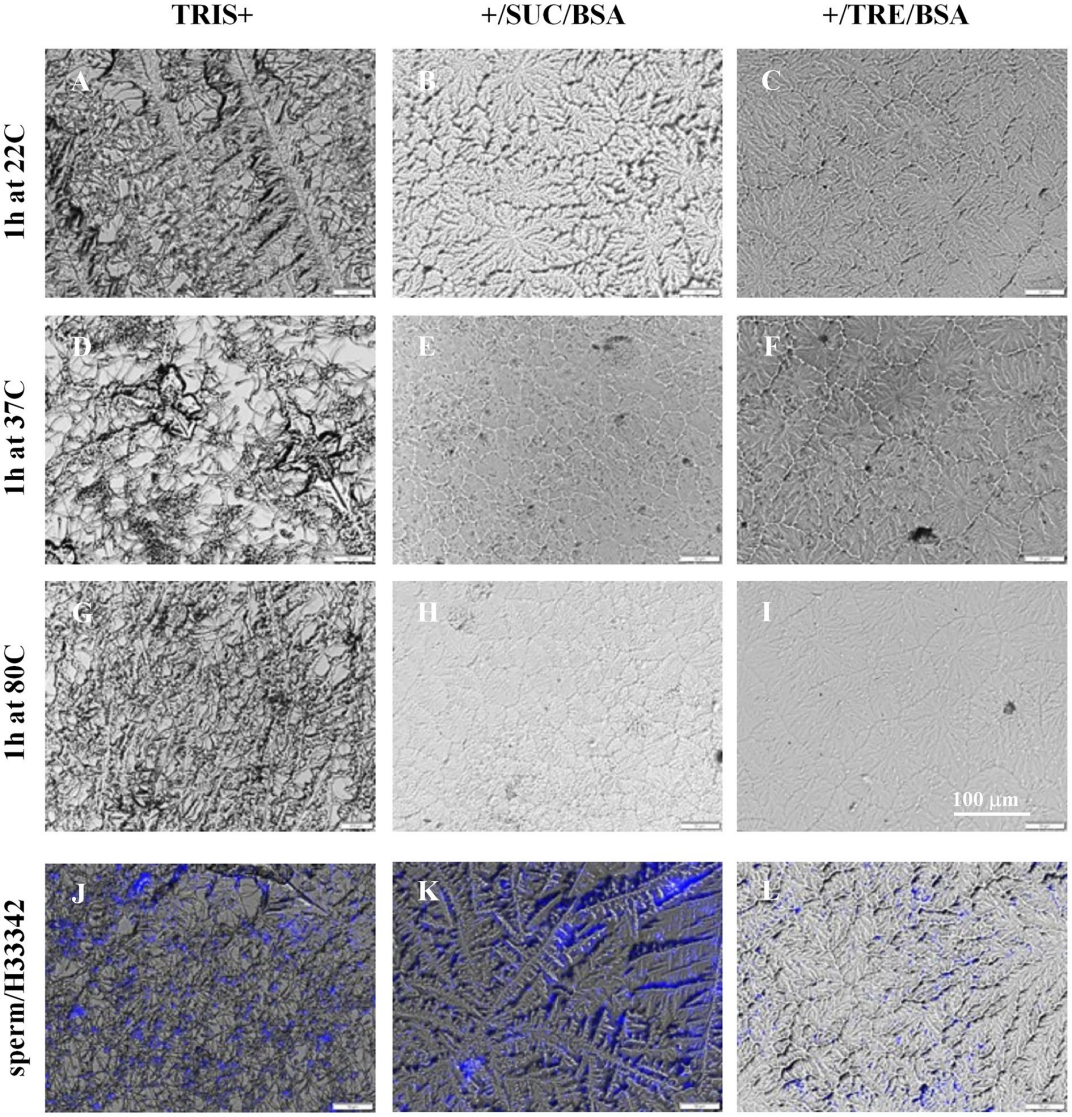
Appearance of different sperm preservation formulations subjected to drying at different temperatures. Preservation formulations were composed of TRIS-buffer, salt and a chelator (TRIS+), and sucrose (SUC) or trehalose (TRE) and albumin (BSA) added or not (**A**,**D**,**G**,**J**: TRIS+, **B**,**E**,**H**,**K**: TRIS+/SUC/BSA, **C**,**F**,**I**,**L**: TRIS + /TRE/BSA). Fifty-µL droplets were dried for 1 h at temperatures ranging from room temperature up to 80 °C (**A**–**C**: 22 °C, **D**–**F**: 37 °C, **G**–**I**: 80 °C); and observed using light microscopy. Dependent on the absence of sugar/albumin and the drying temperature and rate applied, a crystalline or glassy structure is observed. To visualize the sperm nuclei and distribution, for specimens dried at 37 °C, the fluorescent dye Hoechst33342 was applied (**J**,**K**,**L**).

Droplets of 50 μL consisting of TRIS+/sugar/albumin and sperm, were added on glass slides that were maintained at several temperatures. Sperm drying was done using the following concentrations per mL: 100 × 10^6^ sperm, and either no or 1.71 mg sugar [i.e., trehalose, sucrose or glucose] and 1.71 mg albumin (i.e., 1/1 [w/w] sugar/albumin). Weight measurements of the sample versus the drying time indicated that a stable state was attained after 30 and 60 min when drying was done at 37 and 22 °C, respectively (data not shown). The macroscopic appearance of dried specimens was inspected after drying at different temperatures and hence drying rates (Fig. 1). It can be seen that crystal formation and crystal size are dependent on both the drying temperature as well as the composition of the formulation. The dried TRIS+ samples without supplements showed typical crystals in all cases. The smoother appearance of the TRIS+/sugar/albumin formulations, especially after faster drying (i.e., at higher temperatures), is indicative of a glassy state. The incidence of crystal formation appears to be lower for formulations that included trehalose compared to those with sucrose, particularly at lower temperatures. A fluorescent dye that stains sperm nuclei was added to inspect the sperm distribution in dried specimens. Merged light microscopic and fluorescent images illustrate that sperm are evenly distributed within the dried crystalline/glassy structure.

### Accumulation of ROS and sperm DNA damage during dried storage is affected by the storage temperature and presence of disaccharides

**Figure 2:**
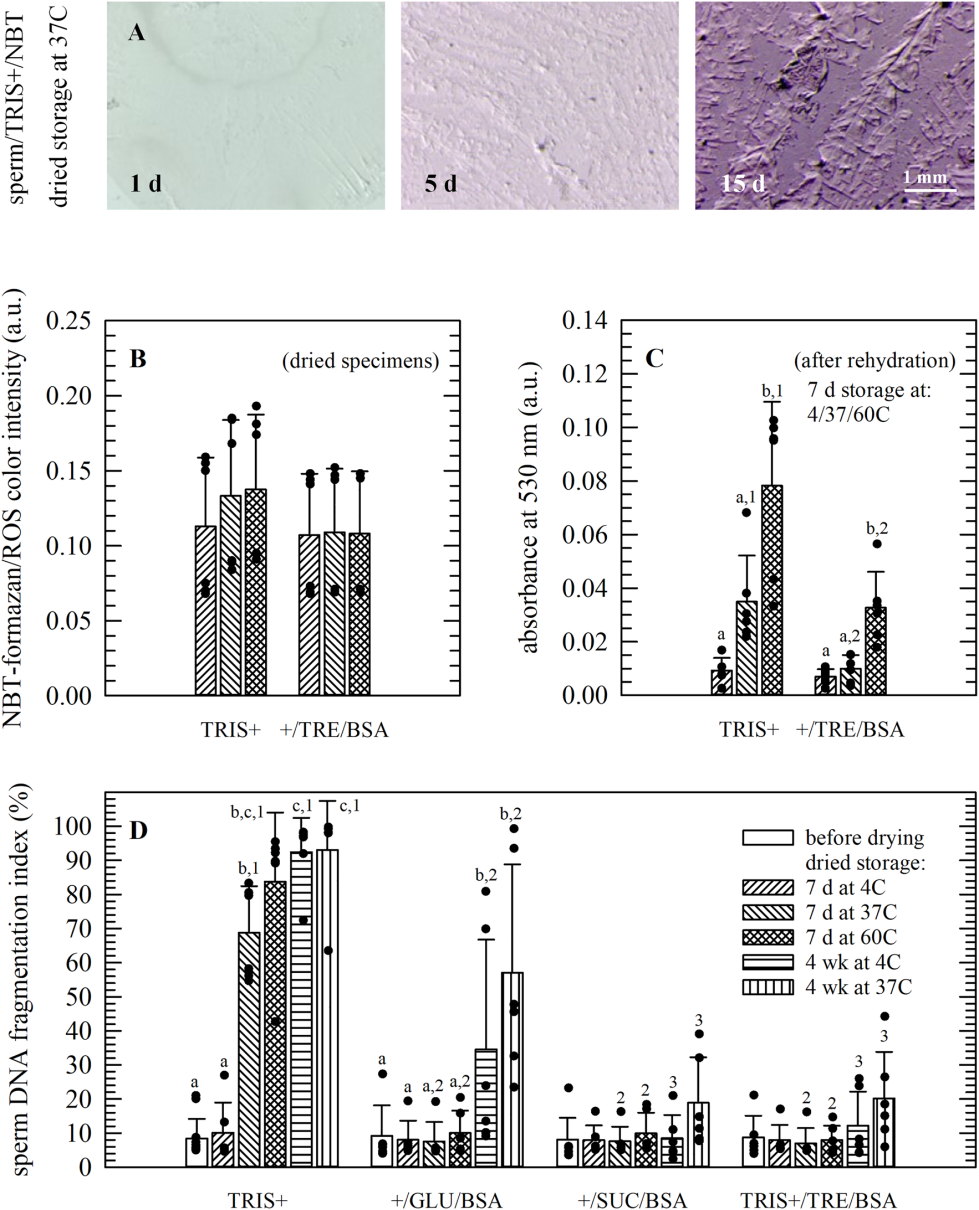
Accumulation of reactive oxygen species (ROS) and changes in sperm chromatin structure (i.e., the DNA fragmentation index/DFI) during dried storage. Specimens were prepared in TRIS+ without supplements (**A**–**D**) as well as TRIS+ supplemented with albumin and trehalose (**B**–**D**), sucrose or glucose (**D**). ROS accumulation was analyzed in specimens stored for 1 week at temperatures ranging from 4‒60 °C (**B**,**C**), while DFI-values were determined during storage for up to 12 weeks (**D**). Panel (**A**) depicts representative images of dried sperm/TRIS+ specimens, supplemented with NBT, that were stored for up to 2 weeks at 37 °C. ROS formation and accumulation is evident as an increased blue/purple coloration due to formazan formation. Formazan formation was quantified for specimens stored for 7 days at 4, 37 and 60 °C; both via image analysis of micrographs from dried samples (**B**) and via spectrophotometric assessments after hydration (**C**). The sperm chromatin structure assay was used for determining DFI-values as a measure for DNA damage during dried storage at different temperatures in different formulations (**D**). Mean values ± standard deviations are presented as determined from three (**B**,**C**) or six (**D**) specimens per treatment, prepared using a split sample approach and semen from different stallions. Statistically significant differences amongst formulations tested and time points of analysis are indicated with, respectively, different letters and numbers.

Accumulation of ROS and oxidative damage in dried specimens (i.e., dried matrix with sperm embedded) was analyzed by supplementing samples with NBT prior to drying. ROS reacting with NBT gives rise to formazan formation, which is evident as a dose-dependent increase in blue/purple coloration (Fig. 2A), especially for samples that were stored at temperatures higher than 4 °C without addition of protective agents (i.e., using TRIS + without supplements). Formazan and ROS accumulation during storage in the dried state increased with increasing storage temperature, which appeared not be the case in specimens supplemented with trehalose/albumin (Fig. 2B; not significant). Spectroscopic assessments after rehydration (i.e., A530-values), of specimens stored for 1 week at 4 or 37 °C, showed a similar trend as obtained via image analysis of the dried state. For specimens stored at 60 °C formazan formation after rehydration was detected both in TRIS + with and without trehalose/albumin, however, values were much lower for the latter (Fig. 2C).

Sperm chromatin structure and DNA fragmentation were also investigated during dried storage. DNA fragmentation index (DFI) values were determined after 1 week storage at temperatures ranging from 4 to 60 °C and after storage for up to 12 weeks at 37 °C. Sperm DNA damage increases with increasing storage temperature and duration in TRIS + formulations (Fig. 2D). DNA damage during storage is prevented when sugar/albumin is added in the preservation formulation. In TRIS + , a sperm DFI-value of 8 ± 6% was determined for fresh samples, whereas after drying and one week storage at 4 and 37 °C DFI-values were found to be, respectively, 10 ± 9 and 69 ± 14%. Presence of albumin and the reducing sugar glucose appeared to be less effective, during storage at 37 °C, with values increasing from 8 ± 6% after 1 week to 35 ± 32 and 57 ± 32% after, respectively, 4 and 12 weeks. When TRIS + was supplemented with albumin and either trehalose or sucrose, sperm DFI-values did not increase during dried storage at 37 °C for up to 1 month (i.e., DFI-values maintained at values ranging from 8 to 12%). Slightly higher DFI-values were determined after 3 months storage (~ 20%).

### Characteristic infrared spectral features of nucleic acids and preservation formulations

**Figure 3:**
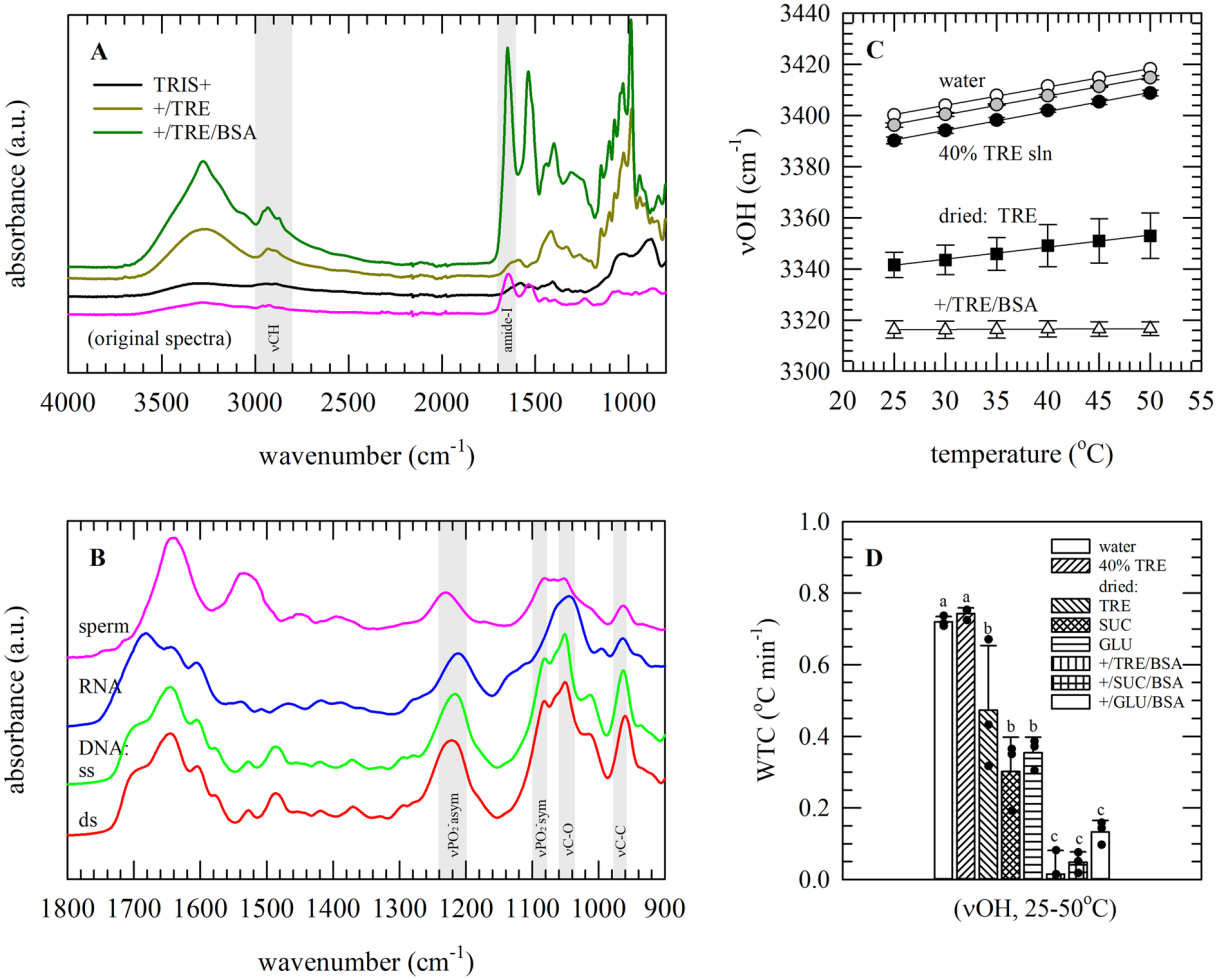
Infrared spectra of different sperm preservation formulations (**A**), as well as pure/isolated nucleic acids and sperm (**B**). In addition, for preservation formulations, spectra were collected at different temperatures and the OH-stretching band position (νOH) was analyzed (**C**,**D**). Full original spectra are shown, of dried specimens, for TRIS+ without supplements (black line), or TRIS+ supplemented with trehalose (+/TRE; dark green line) and albumin (+/TRE/BSA; green line). In addition, a full spectrum of dried sperm in saline is shown (pink line). In panel (**B**), for dried nucleic acids (dsDNA: red line, ssDNA: green line, RNA: blue line) and sperm, the 1800–900 cm^−1^ spectral region is presented; in which specific absorbance bands are assigned. In (**C**), plots on νOH versus the sample temperature are presented; for water (open circles), trehalose solutions (5% TRE: grey circles, 40% TRE: black circles), dried trehalose in a glassy state (TRE: closed squares) and dried preservation formulation/TRIS+ supplemented with trehalose/albumin (+/TRE/BSA: open triangles). Panel (**D**) presents wavenumber temperature coefficient (WTC) values for different hydrated and dried formulations, determined from νOH versus temperature plots in the 25–50 °C temperature range. Average spectra are presented, as well as mean values ± standard deviation, derived from analysis of three specimens. Statistically significant differences amongst formulations are indicated with different letters.

Figure 3A,B shows typical infrared (IR) spectra of dried isolated/pure nucleic acids as well as sperm in TRIS+/preservation formulations. In the 3600–3000 cm^−1^ spectral range, a pronounced νOH stretching vibration band can be found, resulting from water (i.e., in case of hydrated samples), sugars and/or proteins. The CH-stretching region (3000–2800 cm^−1^) mainly arises from CH_3_ and CH_2_ groups of proteins and membrane lipids. CO-stretching and NH-bending vibrations from the protein backbone give rise to the amide-I and -II band at ~ 1650 and ~ 1550 cm^−1^, respectively. In the so-called fingerprint region (1500–900 cm^−1^), a variety of characteristic IR group frequencies can be observed, including specific absorbance bands resulting from proteins and nucleic acids. The presence of trehalose is evident as five characteristic peaks in the 1170–960 cm^−1^ region. Absorbance bands resulting from DNA include the C–C backbone (965 cm^−1^), carbonyl deoxyribose (1050 cm^−1^) and symmetric and asymmetric PO_2_^−^ (1089 and 1235 cm^−1^) stretching vibrations. RNA-specific peaks from the ribose rings can be found at 1125 and 993 cm^−1^.

In contrast with crystalline samples, an amorphous (i.e., glassy, non-crystalline) state is generally characterized by broad absorbance bands, especially in the OH stretching region. The position of νOH and its shift with temperature provide a measure for intermolecular hydrogen bonding interactions. The νOH wavenumber decreases with increasing sugar concentration in hydrated sugar solutions, and further decreases upon drying and addition of albumin (Fig. 3C). In addition, the temperature-dependent shift in νOH (i.e., wavenumber temperature coefficient or WTC-value) is higher for liquid samples as compared to dried amorphous samples, indicating that formation of an amorphous glassy state decreases the thermal expansion of hydrogen bonding interactions (Fig. 3D).

### Drying- and temperature-induced conformational changes in nucleic acids and sperm chromatin, as detected using infrared spectroscopy

**Figure 4:**
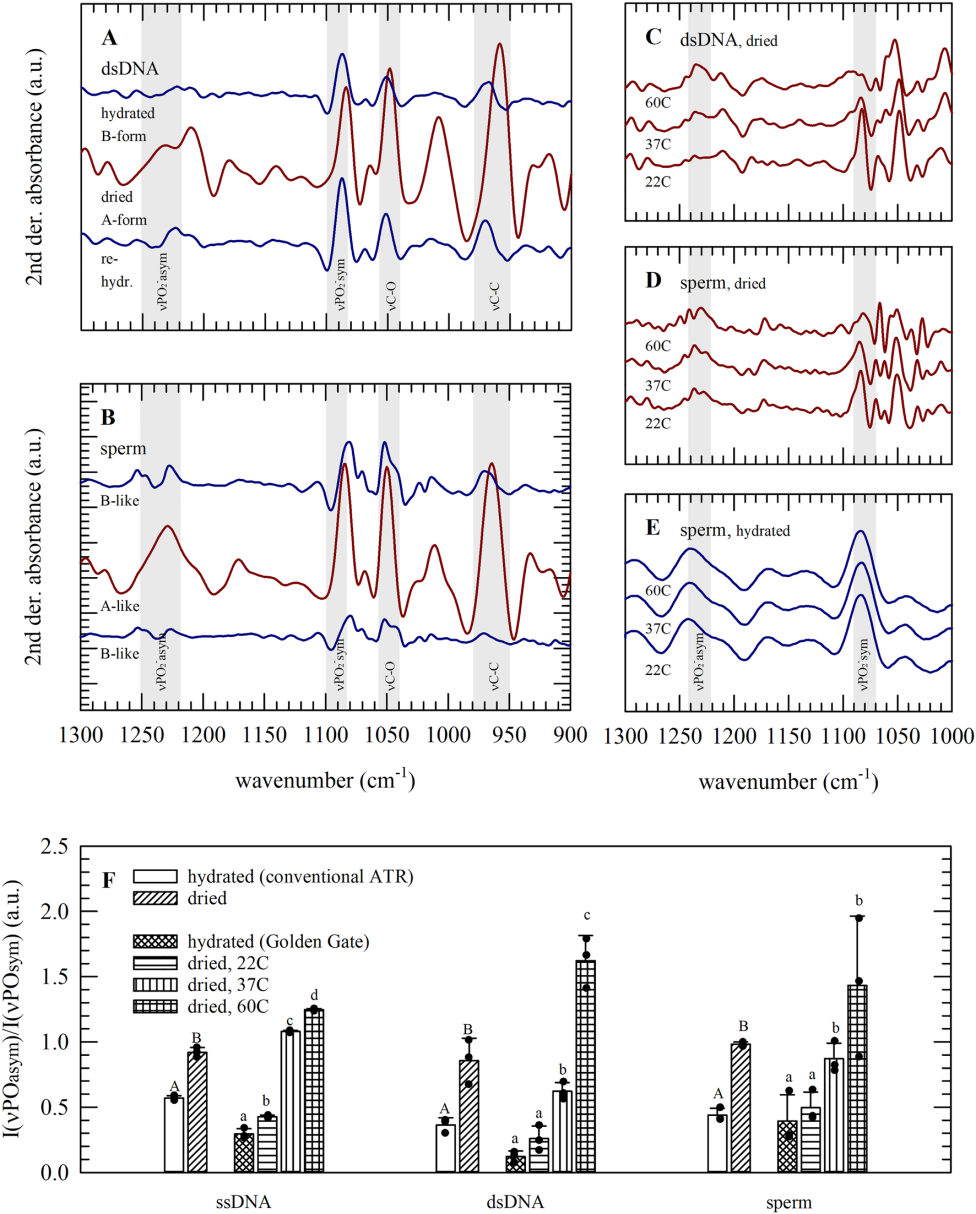
Second derivative infrared spectra (**A**–**E**), and analysis of DNA conformational changes by means of assessment of absorbance band ratios (**F**); for dsDNA (**A**,**C**,**F**), ssDNA (**F**) and sperm samples (**B**,**D**–**F**). Spectra were acquired in the hydrated state, after subjecting specimens to drying, as well as drying-and-rehydration (**A**,**B**), and in the dried state at different temperatures (**C**−**E**,**F**). Characteristic absorbance band regions in which changes from the B- to A-like DNA form appear (i.e., for hydrated and dried specimens, respectively) are indicated. Band intensity ratios of the asymmetric versus symmetric PO_2_^−^ stretching vibration [I(*v*PO_2_^−^_asym_)/I(*v*PO_2_^−^_sym_): Iν1225/Iν1089 and Iν1235/Iν1089 for hydrated and dried specimens, respectively] were derived from normalized second derivative spectra of the fingerprint region; for quantifying drying- and temperature-induced conformational changes (**F**). Average spectra are presented and mean values ± standard deviations, as derived from three specimens. Statistically significant differences amongst treatments are indicated with different letters.

Dehydration causes DNA to undergo a conformational transition from the B-form to the A-form, which is visible as a change in several characteristic absorbance bands. Figure 4 depicts the changes in the position and shape of nucleic acid specific absorbance bands in response to dehydration and rehydration, as well as heating. Upon dehydration, both for pure dsDNA and sperm, the DNA B-to-A-form transition is visible as: a νC–C shift from 970 to 965 cm^−1^, a νC–O shift from 1052 to 1050 cm^−1^, while νPO_2_^−^_asym_ shifts from 1225 to 1235 cm^−1^. These wavenumber shifts appear to be reversible upon rehydration, and spectral features of hydrated/fresh specimens appeared to be similar compared to those of dehydrated-rehydrated specimens. The intensity of νPO_2_^−^_sym_ at 1089 cm^−1^ was found to increase upon dehydration (Fig. 4A,B), while drying at higher temperatures resulted in a decrease in band intensity (Fig. 4C,D). This indicates temperature-induced changes in the DNA backbone and sperm chromatin structure of dried specimens.

Figure 4F shows the band ratio of the asymmetric and symmetric PO_2_^−^ vibration bands for hydrated as well as dried specimens. It can be seen that I(*v*PO_2_^−^_asym_)/I(*v*PO_2_^−^_sym_) increases during the dehydration-induced DNA B-to-A-form transition both for pure/isolated nucleic acids and sperm. In addition, the ratio increases with increasing drying temperatures.

### Spectral analysis of changes in sperm biomolecular structure during dried storage in the absence and presence of trehalose

**Figure 5:**
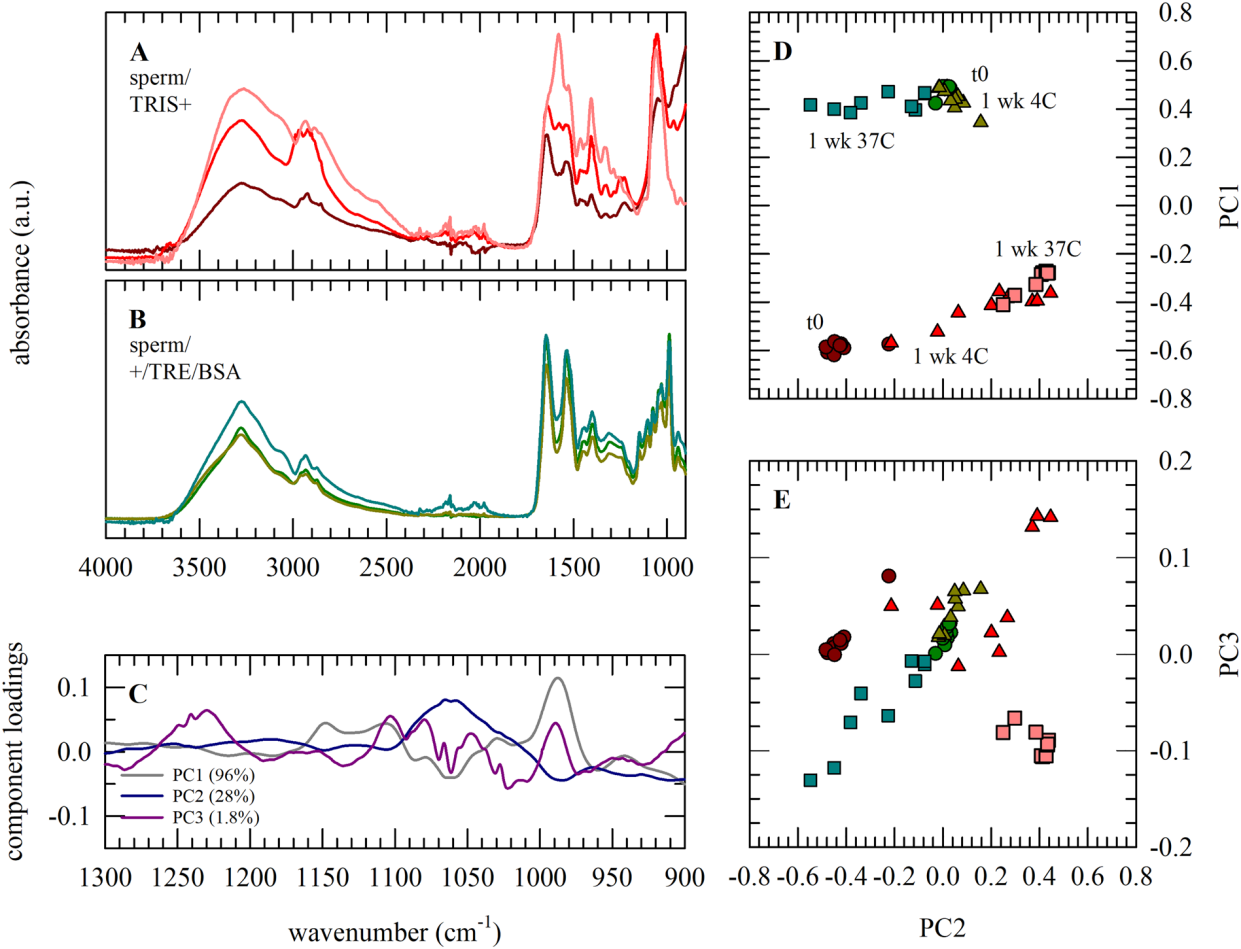
Infrared spectroscopic analysis of sperm samples subjected to drying and storage using different protective formulations and storage temperatures. Specimens were prepared in TRIS+ without supplements (**A**,**D**,**E**; red lines and symbols) as well as TRIS+ supplemented with trehalose and albumin (**B**,**D**,**E**; green lines and symbols). Specimens were analyzed immediately after drying (dark colors, circles), as well as after 1 week dried storage at 4 °C (triangles) or 37 °C (light colors, squares). Full original spectra are shown in panel (**A**,**B**), while the fingerprint region was subjected to PCA (**C**–**E**). The component loadings plots of PC1–3 (**C**) illustrate the spectral regions which appear different amongst formulations used and/or storage conditions. Differences amongst specimens are evident as separation in score plots on PC2 vs PC1 (**D**) and PC3 (**E**). Three specimens were prepared and analyzed per treatment (i.e., two formulations, three time points), using a split sample approach and semen from different stallions (i.e., three stallions). Three spectra were collected for each specimen, eventually resulting in nine spectra per treatment. Average spectra are represented, while all data points are shown in the PCA plots.

Figures 3A,B and 5A,B illustrate that characteristic nucleic acid and trehalose/albumin absorbance bands overlap, which makes it difficult to assign sperm DNA-specific structural changes in spectra taken during storage (i.e., in various formulations). When sperm is dried in TRIS+, various characteristic spectral features change during storage which are possibly associated with sperm damage. In TRIS+ supplemented with trehalose/albumin, however, the overall shape of the spectra does not show obvious changes after storage for up to 1 week at either 4 or 37 °C. The fingerprint region was selected for Principal Component Analysis (PCA; Fig. 5C–E), since it contains DNA-specific bands. A clear separation between sperm prepared and dried in TRIS+ with and without supplements is seen in PC1 versus PC2 score plots. PC1 explains 69% of the observed variance, and has major component loadings around 990 and 1050 cm^−1^. PC2 and PC3 depict 28 and 1.8% of the observed variance, respectively, and are separated by component loading bands at ~ 1089 and 1235 cm^−1^ likely denoting DNA-PO_2_^−^ stretching vibrations. Spectra of sperm dried and stored for different durations in TRIS+ appear as different clusters in the PC2 versus PC3 score plot, indicating storage causes biomolecular changes. Interestingly, specimens supplemented with trehalose/albumin stored for 1 week at 4 °C form a group/cluster together with the samples analyzed directly after drying, whereas samples stored at 37 °C partly diverge from this group. This indicates that the biomolecular composition/structure is preserved in case of drying sperm with trehalose/albumin and dry storage at low temperature.

## Discussion

Reactive oxygen species and DNA damage were found to accumulate in dried stallion sperm samples with increasing storage temperature and storage duration. Oxidative stress and sperm DNA damage (i.e., DFI-values) are known to negatively affect fertility rates^11,39−41^. For mice it has been reported that when dried sperm is used for fertilization, numbers of offspring tend to decrease with increasing storage duration^14,15^. In the current study it is found that for stallion sperm the use of preservation formulations supplemented with disaccharides (i.e., trehalose, sucrose) and albumin counteract oxidative stress and enhance sperm chromatin stability during storage. This can be attributed to scavenging actions neutralizing the damaging effects of reactive oxygen species as well as a drastic decrease in the rate of damaging reactions in the glassy state^32,34^. The increased damage in the presence of the reducing monosaccharide glucose can be explained by Amadori and Maillard (i.e., browning) reactions between glucose and proteins, which even take place in dried samples^19^. Glucose has a free aldehyde group in its open conformation, which can react with amino groups in proteins, resulting in a cascade of reactions and protein modifications. This may result in malfunctioning, e.g., in case proteins have an enzymatic function.

Our microscopic observations and FTIR studies revealed that evaporative drying at elevated temperatures combined with the use of trehalose and albumin facilitated formation of a glassy state, with a strong hydrogen bonding interaction network in which sperm were embedded. For freeze-dried specimens, we previously determined a Tg-value of 41 °C^19^, confirming the presence of a glassy state at 37 °C for such specimens. The glass transition temperature and physical properties of the amorphous state have been described before to correlate with sample stability^28,38,41^. It should be noted that the extracellular and intracellular state are not necessarily the same. To provide intracellular protection, various approaches have been proposed to load mammalian cells with lyoprotective agents like trehalose^5^. Our previous studies revealed that when cells/sperm are subjected to freezing, a dehydration-induced membrane phase transition takes place which facilitates uptake of extracellular trehalose^19,42^, and increased storage stability of freeze-dried specimens^19,28^. It is likely that trehalose also enters the cell during evaporative drying^42^, however, this remains to be determined. Moreover, even though trehalose may have entered the cellular cytoplasm, it may not directly interact with the tightly packed chromatin structure.

In contrast to freeze-drying, drying times under ambient temperature conditions should be kept as short as possible to minimize degradation reactions during the drying process itself. Sperm heat-drying has been applied as a simple approach to accelerate drying^9,43,44^. Proteins in hydrated sperm, however, start to denature around 45–50 °C^29^, so it can be expected that too high drying temperatures cause functionality losses. To further shorten drying times and increase reproducibility, porous filters can be used to decrease the sample vapor pressure and increase the available surface area for drying^45^ and/or microwave-assisted drying^46,47^. Filters, however, may not be suitable for all cell types, particularly not for cells such as sperm that may get trapped inside the filter material.

Similar as seen for pure DNA, it was found here that sperm chromatin underwent a reversible B- to A-DNA transition upon dehydration. In infrared spectra this was evident as a drying induced shift of νPO_2_^−^_asym_ from 1225 to 1235 cm^−1^, and an increase in the intensity of νPO_2_^−^_sym_ at 1089 cm^−1^, in agreement with previous findings^30,31^. In the absence of protective agents, drying and exposure to elevated temperatures resulted in an increase in the asymmetric/symmetric phosphate band intensity ratio value. The increase in I(νPO_2_^−^_sym_)/I(νPO_2_^−^_asym_) likely coincides with a tightening of chromatin packing due to water removal. Others reported that the asymmetric/symmetric phosphate band intensity ratio is also increased by increased protamine-DNA binding^48,49^, and decreases after treating sperm with the disulfide reducing agent dithiothreitol^29^. Non-invasive spectral analysis of sperm can be correlated with morphological characteristics and fertility, which holds great promise in artificial reproduction technologies^50−54^. Infrared and Raman spectroscopy both are vibrational spectroscopy methods that do not require sample labeling. Both methods rely on characteristic molecular group vibrations of endogenous molecules in a sample. Changes in molecular vibrations may denote conformational phase and state changes and/or chemical modifications. FTIR combined with PCA was applied here to reveal molecular damage of dried sperm during storage. The advantage of PCA is that it is based on an entire spectral region rather than on changes in selected specific bands. No storage-related spectral changes were determined by PCA when sperm was dried with trehalose/albumin and stored at refrigerated temperatures. At elevated temperatures, however, PCA revealed spectral changes indicating alterations in biomolecular composition/structure.

Free radical-mediated oxidation has been implicated as the main cause of DNA degradation during storage. DNA packaged in chromatin is more stable than naked DNA, and purified DNA is more stable than DNA in damaged cells^56^. Accumulated reactive oxygen species may directly alter chromatin structure, or transient lipid radical molecules that are formed during lipid peroxidation may cause DNA damage^57^. In sperm the degree of chromatin condensation typically determines sensitivity to DNA damage^29^. It should be noted that there are distinct differences amongst species in chromatin structure, which cause differences in susceptibility for oxidative damage. Damage may only become apparent after pro-longed storage or during storage (or transport) under sub-optimal conditions.

Taken together, evaporative drying of sperm samples provides a simple method to preserve sperm chromatin for genome resource banking. Use of preservation solutions supplemented with trehalose and albumin, and drying done at elevated temperatures, results in the formation of a glassy matrix in which sperm are embedded. This approach appears to counteract the accumulation of reactive oxygen species during dried storage, which in turn plays a role in preventing the accumulation of sperm DNA damage. Furthermore, it was shown that FTIR spectroscopy combined with PCA provides a powerful non-invasive tool to detect sperm biomolecular structure. It allows for monitoring possible alterations in sperm chromatin structure during storage, for quality evaluation prior to use for artificial reproductive technologies.

## Materials and methods

### Semen collection and processing

All procedures involving animals were carried out in accordance with German animal welfare legislation and approved by the Lower Saxony State Office for Consumer Protection and Food Safety (33.12-42502-05-17A225).

Semen was collected from stallions of the Hanoverian warmblood breed that were held at the Unit for Reproductive Medicine at the University of Veterinary Medicine Hannover as well as the National Stud of Lower Saxony in Celle, Germany. Animals were kept and semen collection and processing were done according to European regulations and animal care and use protocols. The above mentioned institutions are registered and licensed as artificial insemination centers, according to regulations given by the European Union (council directive 92/65/EEC). For the here described studies, aliquots from routine semen collections were used, which were performed for the commercial artificial insemination program of the stud. Semen was collected using an artificial vagina and breeding phantom (both model ‘Hannover’; Minitüb, Tiefenbach, Germany), while a filter was used to remove the gel portion. The sperm concentration was determined using a photometer (Minitüb), where after semen was diluted with at least an equal volume of INRA-82 skim milk extender^58^ of 37 °C. Sperm samples were centrifuged (600×*g*, 10 min), the supernatant was removed, and the sperm pellet was resuspended with fresh diluent to 100 × 10^6^ sperm mL^−1^. INRA-82 had a pH of 6.8–7.0 and osmolality of 300–330 mOsm kg^−1^; and was prepared by mixing equal volumes of commercial 0.3% ultra-heat-treated skim milk and glucose saline solution (50 g L^−1^ glucose monohydrate, 3.0 g L^−1^ lactose monohydrate, 3.0 g L^−1^ raffinose pentahydrate, 0.5 g L^−1^ sodium citrate dihydrate, 0.82 g L^−1^ potassium citrate monohydrate, 9.52 g L^−1^ HEPES, 1.0 g L^−1^ penicillin, 1.0 g L^−1^ gentamycin).

### Sperm drying

TRIS+ (10 mM TRIS–HCl, 1 mM EDTA, 150 mM NaCl, pH 8.0) was used as diluent for drying of sperm^19,59^. After centrifugation, sperm was diluted to 200 × 10^6^ sperm mL^−1^ in TRIS+, followed by dilution with an equal volume of TRIS+ without supplements or TRIS+ supplemented with two-fold the desired final concentration of protective agents. This resulted in 100 × 10^6^ sperm mL^−1^, in TRIS+, and either no or 1.71% (w/v) trehalose (TRE), sucrose (SUC) or glucose (GLU) and 1.71% (w/v) bovine serum albumin (BSA; fraction V) added at a 1/1 weight ratio (i.e., w/w, sugar/albumin). In case of trehalose and sucrose, 1.71% equals 50 mM, whereas 1.71% glucose equals 100 mM. Drying of sperm samples was done by adding 50 µL droplets onto glass microscope slides, which were kept at 37 °C on a temperature-controlled plate for 30 min unless otherwise stated. Drying was done under ambient conditions, at a relative humidity of 35–55%, and the final sample water content was less than 0.1 g water per g dry weight.

Drying kinetics and sample water contents were determined gravimetrically, by weighing specimens using a microbalance (i.e., slides with droplets sperm sample added) versus the drying time. Weights of the actual sperm samples were determined by subtracting the weights of the slides as determined before adding droplets. Sample water contents (g H_2_O/g dry weight) were calculated by subtracting the sample weights after drying from the sample weight after incubating the sample overnight at 80 °C (i.e., after removal of all residual water), and dividing this by the dry weight.

Sperm samples were rehydrated directly after drying or stored as dried specimens. The latter was done after sealing in plastic bags to limit water absorption during storage. Dry specimens were stored for 1 week at temperatures ranging from 4‒60 °C, in a fridge or incubator, and for up to 3 months in an incubator set at 37 °C. For spectroscopic assessments, dried specimens were analyzed directly after preparing or storage at specific conditions. For the sperm chromatin structure assay, samples were rehydrated, frozen, and stored in liquid nitrogen or at − 80 °C until analysis. Rehydration was done by adding distilled water directly onto the dried samples, where after the plastic micropipette tip was used for gentle scratching to recover all the sample. Typically, material originating from 2 to 3 dried droplets per slide was pooled.

### Microscopic assessments of sperm dried in various formulations, and quantification of ROS/NBT-formazan formation in specimens stored at different temperatures

Directly after drying at different temperatures, the macroscopic appearance of TRIS+ formulations with embedded sperm was evaluated, at a 10 × 20 magnification, using an inverted microscope (Olympus, Hamburg, Germany) equipped for Hoffman modulation contrast and fluorescence observations. Sperm were stained by supplementing specimens with 150 µg mL^−1^ Hoechst33342, which exhibits blue fluorescence upon intercalating within DNA.

Nitroblue tetrazolium (NBT) was used to visualize and quantify accumulation of reactive oxygen species (ROS) in dried specimens during storage. NBT forms formazan upon reacting with ROS/superoxide which is evident as a blue-colored precipitate^60,61^. Sperm specimens were prepared in TRIS+ supplemented with 0.1 mg mL^−l^ NBT, and dried for 30 min at 37 °C as described above. Dried specimens were stored for 7 days at temperatures ranging from 4‒60 °C, where after they were inspected microscopically. Micrographs were collected using a stereomicroscope (Olympus) with camera setup, at a 10 × 6.3 magnification, using the same settings and exposure time for all samples. ImageJ software (National Institutes of Health, Bethesda, MD, USA) was applied to convert micrographs to 8-bit images, set a global calibration, and deriving gray values as a measure for the blue/purple coloration intensity of the specimens. For spectrophotometric analysis an ordinary UV/visible spectrophotometer (Jenway) was used, as described before^61^. After rehydration, samples (2 × 50 µL) were transferred to a microtube and 100% DMSO was added (900 µL). After vortexing for 30 s, and centrifugation (13,000×*g*, 1 min), the supernatant was transferred into a 1 mL-cuvette and the absorbance at 530 nm was recorded. As blank, DMSO/diluent without recovered sperm sample was measured.

### Analysis of sperm chromatin structure

The sperm chromatin structure assay (SCSA) was used to evaluate chromatin integrity as previously described in detail^62^. In short, sperm was diluted in TNE (10 mM TRIS–HCl, 150 mM NaCl, 1 mM Na_2_EDTA, pH 7.4), acid/detergent solution (80 mM HCl, 150 mM NaCl, 0.1% Triton X-100, pH 1.2) was added, followed by vortexing for 30 s, and addition of acridine orange staining and neutralization solution (4.5 μg mL^−1^ acridine orange, 150 mM NaCl, 37 mM citric acid, 126 mM Na_2_HPO_4_, 1 mM Na_2_EDTA, pH 6.0). Samples were placed on ice and analyzed after 3 min; using a DAKO Galaxy flow cytometer (Dako Cytomation GmbH, Hamburg, Germany), equipped with a 488 nm laser for excitation and 538/23 and 590/25 nm band pass filters for detecting, respectively, green and orange fluorescence. Acridine orange fluoresces green and orange in case of presence of, respectively, double- and single-stranded DNA. Sperm were selected based on their forward and side scatter properties, and DNA fragmentation index (DFI) values were determined by quantifying sperm populations showing differences in green and red fluorescence as described in detail elsewhere^62^. DFI values were derived using Kaluza software (Beckman-Coulter, Krefeld, Germany).

### Fourier transform infrared spectroscopic measurements

Fourier transform infrared spectroscopy (FTIR) was used to study drying- and temperature-induced conformational molecular changes in pure/isolated nucleic acids and stallion sperm in situ, as well as preservation formulations. A Nicolet iS5 FTIR spectrometer (Thermo-Fisher, Waltham, MA, USA) was used, with Omnic software. The device was equipped with an attenuated total reflection (ATR) accessory with diamond/ZnSe crystal (i.e., for use at room temperature) and setup for acquisition of transmission spectra. Transmission spectra were collected using a temperature-controlled sample holder connected to a heater device (Harrick Scientific Products, Pleasantville, NY, USA). For acquisition of ATR spectra at different temperatures, a Specac Golden Gate accessory with a monolithic diamond ATR crystal and a heater device (Specac Limited, Orpington, Kent, UK) was used. A T-type thermocouple (Fluke, Everett, WA, USA) was used for measuring the actual sample temperature. Spectra were acquired using an automatic CO_2_/H_2_O vapor correction algorithm, and the following acquisition parameters: 4 cm^−1^ resolution, 8 co-added interferograms, 4000–650 cm^−1^ wavenumber range.

### Spectral analysis of nucleic acids and sperm samples

Nucleic acids that were purchased included: double-stranded (ds) DNA from herring testes, single-stranded (ss) DNA from salmon testes, and RNA from baker’s yeast (Merck KGaA, Darmstadt, Germany). All nucleic acids were prepared as 10 mg mL^−1^ solutions in water. Stallion sperm samples (diluted in INRA-82, stored at 4 °C for up to 1 day), were centrifuged (30 s at 13,000×*g*, twice) for washing and resuspension in saline (0.9%, w/v, NaCl) prior to use. In addition, for spectral analysis of changes in sperm biomolecular structure during dried storage, samples were analyzed that were prepared as described above (i.e., sperm diluted in TRIS+ with or without trehalose/albumin added, 50 µL droplets on glass slides, dried at 37 °C during 30 min before storage at 4 or 37 °C).

Hydrated specimens (10 µL) were sandwiched between two CaF_2_ windows, mounted in the temperature-controlled sample holder, and spectra were acquired at specific temperatures during heating from room temperature (i.e., ~ 22 °C) up to 60 °C. For analysis of drying kinetics at different temperatures, 10 µL sample was added onto the Golden Gate ATR crystal, after setting a specific temperature (i.e., ~ 22, 37 or 60 °C). Spectra were collected every 180 s during 30 min or until no further spectral changes were recorded. Thereafter, specimens were rehydrated by adding 10 µL water, and spectra were acquired to assess reversibility of the observed biomolecular conformational changes. Sperm samples that were subjected to drying on glass slides (50 µL droplets, 30 min at 37 °C) and storage in different formulations (i.e., in TRIS+ with and without trehalose and albumin, for up to 7 days at 4 and 37 °C), were analyzed at room temperature. Dried samples were gently pressed on the ATR crystal using the ATR force arm to facilitate contact.

The fingerprint region (1500–900 cm^−1^) was selected for spectral analysis of changes associated with drying- and temperature-induced conformational changes in nucleic acids and sperm. This region contains the anti-symmetric and symmetric PO_2_ stretching vibrations at, respectively, ~ 1240–1230 and ~ 1089 cm^−1^. Dependent on the hydration state and DNA conformation, the absolute peak position and shape of these absorbance bands changes^26^. Second derivative spectra were calculated using a 21-point smoothing factor and the Savitzky-Golay method to better resolve the absorbance bands that were used for determining band intensity ratios. In addition, principal component analysis (PCA) of the fingerprint region was performed using MATLAB (Mathworks, Natick, MA, USA) to reveal complex differences amongst spectral patterns. For PCA, original spectra were vector-normalized and covariance matrix data analysis was used as previously described^63^.

### Spectral analysis of preservation formulations

FTIR was also used to investigate hydrogen bonding interactions of dried preservation formulations. More specifically, temperature-dependent changes in the νOH band position at ~ 3300 cm^−1^ were investigated^38,41,63^. Pure sugar solutions (10 mg mL^−1^) were prepared in phosphate buffered saline (137 mM NaCl, 2.7 mM KCl, 10 mM Na_2_HPO_4_, 1.8 mM KH_2_PO_4_, pH 7.4), whereas TRIS+ formulations were prepared as describe above. Dried specimens were prepared for analysis by depositing 20 µL solution on a CaF_2_ window that was kept at 37 °C for 60 min. Thereafter, specimens were returned to room temperature and an O-ring/spacer and CaF_2_ window were added for mounting the sample in the temperature-controlled sample holder. Transmission spectra were acquired at 5 °C intervals during heating from 25 to 50 °C. The 3700–3000 cm^−1^ spectral region of the spectra obtained at varying temperatures was selected and the νOH band position was determined as the average of the spectral positions at 80% of the full peak height.

### Statistical analysis

Flow cytometric analyses were done using 6 different ejaculates originating from different stallions, whereas spectroscopic analysis were done using 3 different ejaculates originating from different stallions. For all cases, average data are presented, as well as all values measured. All comparisons/figures presented were derived by testing all presented treatments on the same ejaculates (i.e., using a split sample approach). Statistical analysis was performed using Sigmaplot software (version 13.0; Systat Software GmbH). Normal distribution of data was tested using the Shapiro–Wilk test, and differences among treatment groups were evaluated using one way analysis of variance (ANOVA) followed by Tukey’s multiple comparisons test. Differences were considered to be statistically significant when p < 0.05.
